# Gastrointestinal Hemorrhage in Patient with Granulomatosis with Polyangitis

**DOI:** 10.1155/2021/9921361

**Published:** 2021-10-26

**Authors:** Nikhil Madan, Vipul Patel

**Affiliations:** Division of Pulmonary and Critical Care Medicine, Department of Medicine, Newark Beth Israel Medical Center, New Jersey 07112, USA

## Abstract

Granulomatosis with polyangitis (GPA) is characterized by a necrotizing granulomatous vasculitis of small arteries and veins. It most commonly affects the upper and lower respiratory tract and kidneys. However, other organs including the gastrointestinal tract can be affected. Gastrointestinal manifestations of GPA are rare and can include ischemia, bowel infarction, and perforation. Hemorrhage is an extremely rare presentation of GPA. We present a case of a woman with GPA and pulmonary renal syndrome on treatment who presents with severe gastrointestinal hemorrhage.

## 1. Introduction

Granulomatosis with polyangiitis (GPA) previously known as Wegner's granulomatosis is a primary systemic vasculitis involving the small- and medium-sized vessels. It has an incidence of 5-10 cases per million and equally affects men and women. It is characterized by granulomatous and necrotizing inflammation mainly affecting mainly the respiratory system and kidneys [1]. It can however affect any other organ system. Gastrointestinal involvement is rare with GPA, around 5-11% involvement [2]. Both small and large bowels can be involved causing a serious and potentially fatal complication of GPA.

## 2. Case History

A 58-year-old woman with history of GPA was admitted with melanotic stools. Her GPA history is as follows. She was diagnosed a few months prior to the current presentation with SOB and renal failure and was found to have pulmonary renal syndrome. She underwent serological testing and a kidney biopsy which confirmed the diagnosis of GPA, and she was started on high-dose pulse steroids and Cytoxan. She was also started on hemodialysis for acute renal failure. She was discharged on oral prednisone and Cytoxan but was noted to be pancytopenic shortly after and her Cytoxan was stopped. After her Cytoxan was stopped, she developed significant pulmonary symptoms within a month and she presented back with a cough and shortness of breath. She was then noted to have ground glass opacities on her computerized tomography (CT) scan of the chest and also noted to have a pulmonary embolism (PE) in the left lung. She developed worsening hemoptysis, and her anticoagulation was stopped, and an inferior vena caval (IVC) filter was placed. She was restarted on Cytoxan and underwent plasmapheresis for a total of 5 days.

**Figure 1:**
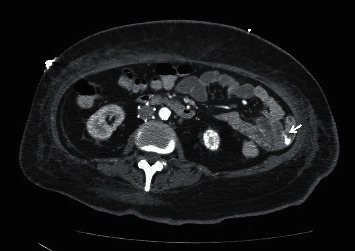
CT angiogram of the abdomen showed active bleeding in the jejunum (arrow).

**Figure 2:**
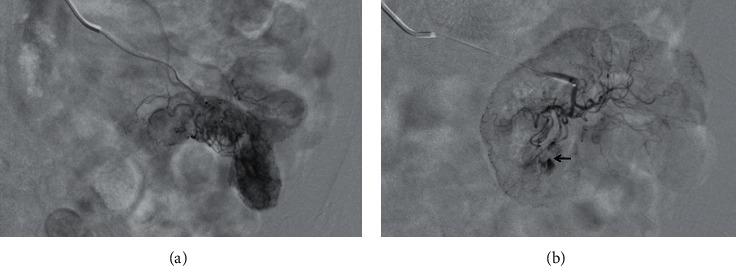
(a) Mesenteric angiogram showed bleeding from the jejunal branch of the superior mesenteric artery (arrow). (b) Late images of mesenteric angiogram showed collection of blood in the loop of the jejunum suggestive of active bleeding.

**Figure 3:**
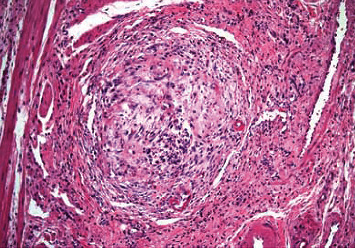
Histopathological examination of the intestine showed granulomatous vasculitis of submucosal vessels.

**Figure 4:**
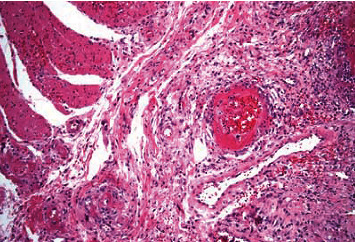
Fibrinoid necrosis of the vascular wall and scattered luminal thrombi present on histopathological examination.

She continued to have melanotic stools despite being off anticoagulation for several days and required multiple red blood cell transfusions. She underwent an upper endoscopy, followed by a colonoscopy and push enteroscopy which were all reported normal and could not identify the source of her bleeding. Her hemoglobin was as low as 6.5 g/dL with continued need for blood transfusions. She underwent a CT angiogram which showed active hemorrhage from the superior mesenteric artery (SMA) (Figure 1). A mesenteric angiogram showed bleeding from the jejunal branch of the superior mesenteric artery (Figure 2(a)). Late images showed collection of blood in the loop of the jejunum (Figure 2(b)). Coil embolization of two branches of SMA was performed. She continued to have ongoing bleeding despite the embolization and underwent exploratory laparotomy with small bowel resection and end-to-end anastomosis. A histopathological exam showed granulomatous vasculitis of the submucosal vessels, fibrinoid necrosis of the vascular wall, and scattered luminal thrombi along with multiple foci of mucosal ulceration and ischemia (Figures 3 and 4). These findings were consistent with GPA involving the small intestine. The patient was started on aggressive treatment with rituximab along with corticosteroids and plasmapheresis. During the hospital course, the patient developed multiple abdominal abscesses in the left lower quadrant and developed septic shock with multiorgan failure. She was placed on mechanical ventilation and started on broad spectrum antibiotics. She continued to have GI bleeding requiring multiple blood transfusions. After a prolonged ICU stay, the patient was noted to have massive intracerebral hemorrhage with herniation. She was declared brain dead and passed in the ICU.

## 3. Discussion

According to the American College of Rheumatology, GPA is defined by the presence of at least two of the four criteria: (1) nasal or oral inflammation; (2) abnormal chest radiograph with the presence of nodules, fixed infiltrates, or cavities; (3) urine sediment with hematuria or red cell cast; and (4) granulomatous inflammation on biopsy within an artery or in the perivascular area of an artery or arteriole.

GI tract involvement in GPA is less common about 10-24% and often detected on autopsy [3]. In a study by Pagnoux et al., looking at 62 patients with GI involvement in small- and medium-sized vessel vasculitis, it was noted that abdominal pain was the most common symptom (97%) followed by nausea and vomiting (34%), diarrhea (27%), and hematochezia and melena (16%) [4]. GPA can affect various sites within the GI tract and can cause esophageal, gastroduodenal and colorectal ulcers, bowel perforations, GI ischemia and infarction, appendicitis, cholecystitis, and acute pancreatitis [4–9]. GPA very commonly affects multiple GI sites, most commonly affecting the small bowel. GI involvement occurs within the first two years of diagnosis [10]. It affects males more than females. Endoscopic diagnosis can be challenging as specimens most commonly show nonspecific inflammation and ulcerations. This is thought to be due to the superficial nature of the biopsies and deeper location of the GPA affected small- and medium-sized vessels in the submucosa [11]. In literature, it is suggested that the use of corticosteroid therapy may be responsible for the development of intestinal manifestations. Irrespective of GI involvement, immunosuppressive therapy should be used. After induction therapy, maintenance with azathioprine or mycophenolate should be used. Surgical intervention may be necessary for GI bleeding, perforation, or gall bladder involvement [12].
